# The Neonicotinoid Insecticide Imidacloprid Disrupts Bumblebee Foraging Rhythms and Sleep

**DOI:** 10.1016/j.isci.2020.101827

**Published:** 2020-11-20

**Authors:** Kiah Tasman, Sean A. Rands, James J.L. Hodge

**Affiliations:** 1 School of Physiology, Pharmacology and Neuroscience, University of Bristol, Biomedical Sciences Building, University Walk, Bristol, BS8 1TD, UK; 2 School of Biological Sciences, University of Bristol, Life Sciences Building, Tyndall Avenue, Bristol, BS8 1TQ, UK

**Keywords:** Ecology, Biological Sciences, Zoology, Animal Physiology, Behavioral Neuroscience

## Abstract

Neonicotinoids have been implicated in the large declines observed in insects such as bumblebees, an important group of pollinators. Neonicotinoids are agonists of nicotinic acetylcholine receptors that are found throughout the insect central nervous system and are the main mediators of synaptic neurotransmission. These receptors are important for the function of the insect central clock and circadian rhythms. The clock allows pollinators to coincide their activity with the availability of floral resources and favorable flight temperatures, as well as impact learning, navigation, and communication. Here we show that exposure to the field-relevant concentration of 10 μg/L imidacloprid caused a reduction in bumblebee foraging activity, locomotion, and foraging rhythmicity. Foragers showed an increase in daytime sleep and an increase in the proportion of activity occurring at night. This could reduce foraging and pollination opportunities, reducing the ability of the colony to grow and reproduce, endangering bee populations and crop yields.

## Highlights

• Field-relevant concentrations of imidacloprid disrupt bumblebee foraging rhythms
• Drugged foragers are inactive for more of the day and more active at night
• Neonicotinoid-treated foragers are more arrhythmic
• These effects occurred in naturalistic 12 h light:12 h dark light cycles

## Graphical Abstract

**Figure:**
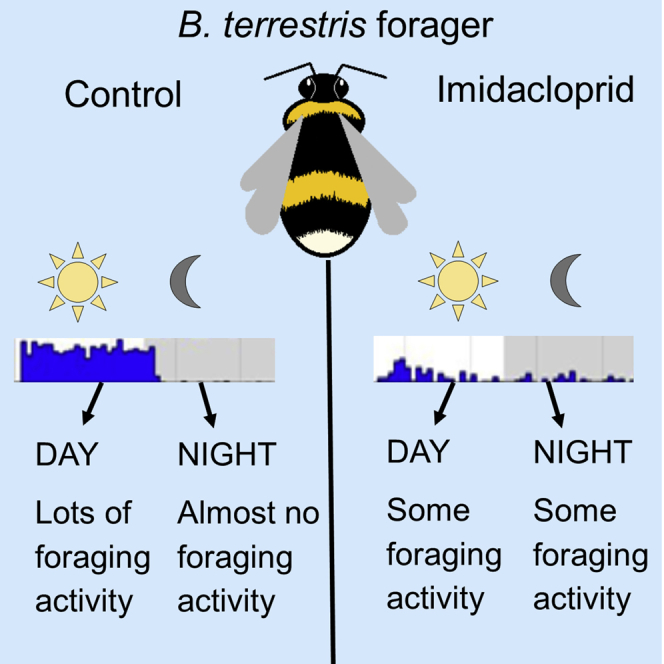

## Introduction

Bumblebees are a diverse and important group of pollinators and are major pollinators of both crops and wildflowers. Of the five most important crop pollinators in Europe, three are bumblebees (Nieto et al., 2014). Many crops are particularly reliant on bumblebee pollination (Willmer et al., 1994; De Luca and Vallejo-Marín, 2013), and crop pollination in Europe is worth over €22bn per annum and is essential for food security (Nieto et al., 2014). Unfortunately, despite their ecological and economic value, bumblebees face dramatic population losses, with 46% of species in Europe in decline and 24% threatened with extinction (Nieto et al., 2014). Due to crop losses to insect pests, demand for insecticides remains high (Casida and Durkin, 2013; Popp et al., 2013). The most common insecticides worldwide are neonicotinoids, global sales of which are worth US$1 billion/year (Popp et al., 2013; Casida, 2018). Neonicotinoids are agonists of nicotinic acetylcholine receptors (nAChR), the main neurotransmitter system in the insect nervous system, and they share target site cross-resistance (Matsuda et al., 2020). They were branded safe compared with their predecessors because they do not act on mammalian nAChRs (Casida, 2018; Matsuda et al., 2020). However, before their introduction to market, sublethal effects were not fully identified in beneficial insects, for which neonicotinoids have proven potent neurotoxins.

Most research on the effects of neonicotinoids on pollinators has used the honeybee, *Apis mellifera* (Blacquière et al., 2012). However, honeybees and bumblebees show differential responses to neonicotinoids, with bumblebees potentially experiencing a higher risk (Cresswell et al., 2012; Stoner, 2016; Gradish et al., 2018). This highlights the importance of increasing the diversity of pollinators studied to determine the ecological consequences of neonicotinoid use. Concentrations as low as 1 μg/L (or 1 part per billion [ppb]) imidacloprid can cause reduced foraging motivation in *Bombus terrestris* (Lämsä et al., 2018). A dose of 6 ppb imidacloprid has been shown in laboratory studies to cause lethargy within the nest (Crall et al., 2018), and cause long-term effects on nest growth and queen production in the field (Whitehorn et al., 2012). This has been replicated in a large-scale study in Sweden, which looked at the effects of neonicotinoid seed coating on wild bees in the field and found reduced colony growth and reproduction in bumblebees (Rundlöf et al., 2015). Neonicotinoids have high solubility and persistence in the environment (Wood and Goulson, 2017), meaning insects are still at risk of exposure, despite the current European Union ban on imidacloprid.

Due to the abundance and importance of nAChRs in the insect central nervous system, the potential sub-lethal effects of neonicotinoids are very broad. Their effect on many behaviors vital to pollination, such as circadian rhythms and sleep, are still unknown. The circadian clock is integral to pollinator foraging efficiency as flower opening, scent release, and nectar production are dependent on time of day (Bloch et al., 2017; Willmer and Stone, 2004). Neonicotinoids have already been shown to have a detrimental effect on pollination, with thiamethoxam exposure of caged *B. terrestris* resulting in reduced pollination of apple trees and fewer seeds in the fruit (Stanley et al., 2015). Circadian rhythms also affect other behaviors, such as sleep, caring for offspring, and learning, with honeybees learning novel, rewarding odors better in the morning (Lehmann et al., 2011; Bloch et al., 2017). This helps them find new foraging patches, as most flowers are nectar-rich in the morning (Bloch et al., 2017).

In *Drosophila*, circadian entrainment (synchronicity within the clock and communication between the light-sensing organs and the central clock) is reliant upon nAChR signaling (Helfrich-Förster et al., 2002; McCarthy et al., 2011; Sheeba et al., 2008), as are the post-synaptic mushroom body (MB) output neurons that regulate sleep (Barnstedt et al., 2016; Palmer et al., 2013). Given the similarity in nAChR expression in the brains of *Drosophila* and the honeybee, this suggests that the clock and sleep of bees may be affected by neonicotinoid exposure (Dupuis et al., 2012). The bee central clock neurons also share similarities with those of *Drosophila*. In *Drosophila*, honeybees, and bumblebees, there are bundles of lateral and dorsal clock neurons, including a set of lateral neurons that express the neuropeptide pigment dispersing factor (PDF) (Beer et al., 2018; Weiss et al., 2009; Lin et al., 2004). Both PDF and nAChR expressing lateral pacemaker clock neurons with extensive branching patterns are also present in other insects, including crickets and cockroaches (Weiss et al., 2009; Numata et al., 2015; Baz el et al., 2013; Helfrich-Förster et al., 1998), demonstrating that clock circuitry is well conserved across insects. In the honeybee, in which the PDF neurons have been well mapped, they have been shown to project to brain regions such as the visual circuitry, *pars intercerebralis* and *pars lateralis* (Beer et al., 2018), both of which control locomotor activity and sleep in *Drosophila* (King and Sehgal, 2018). Furthermore, honeybee clock neurons are important for the sun-compass pathway (Beer et al., 2018), a vital navigational tool allowing the communication of resource location via the waggle dance (von Frisch, 1967). The clock also dictates the timing of sleep, which is required for many vital physiological processes including memory consolidation and synaptic homeostasis (Fisher et al., 2013; Zwaka et al., 2015; Beyaert et al., 2012; Donlea et al., 2009; Stern, 2018; Bushey et al., 2015). Furthermore, sleep timing in bumblebees is important for round-the-clock care of offspring (Nagari et al., 2019). Therefore, circadian and sleep disruption is likely to have detrimental effects to the pollination services, behavior, and fitness of beneficial insects. Due to the importance of nAChR signaling in the insect clock and sleep centers, we hypothesized that neonicotinoids would disrupt bee rhythmicity and sleep. We therefore tested the effect of field-relevant concentrations of imidacloprid on *B. terrestris* foragers.

## Results

The activity of isolated *B. terrestris* foragers in individual tubes was measured using the locomotor activity monitor to assess behavior in both 12 h:12 h light:dark (LD) conditions and constant darkness (DD), (see Supplementary Information, Transparent Methods). Rhythmicity under LD conditions was studied as this provides a more naturalistic reflection of how day/night behavior may be affected in the field and also allows sleep to be investigated. The removal of light cues under constant conditions in DD reveals the endogenous circadian rhythm and clock function.

### Field-Relevant Concentrations of Imidacloprid Affects Rhythmicity in Isolated Bumblebee Foragers in 12 h:12 h Light:Dark Conditions

**Figure 1:**
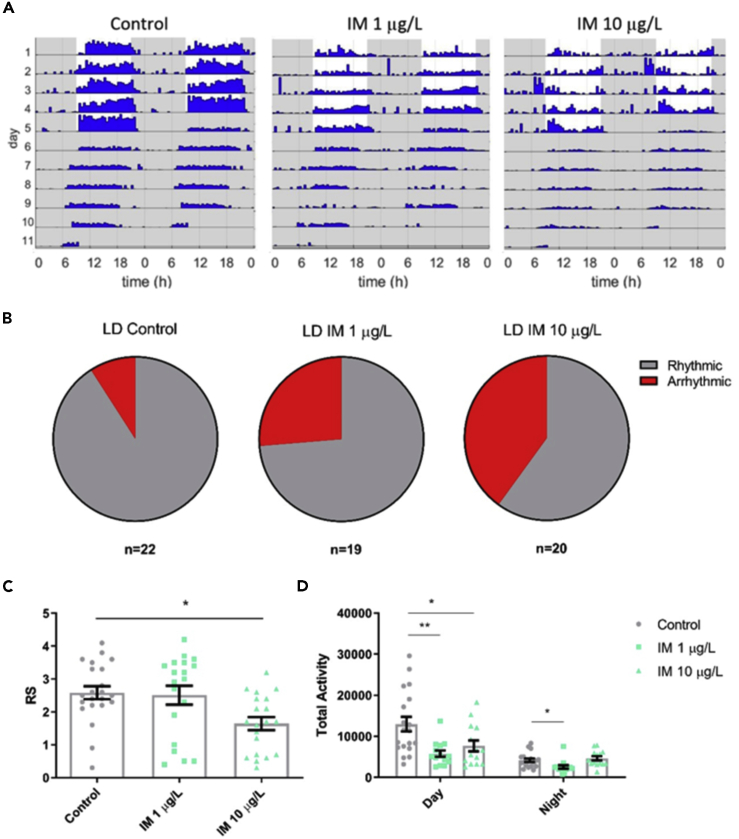
Field-Relevant Concentrations of Imidacloprid Affects Rhythmicity in Isolated Bumblebee Foragers in 12 h:12 h Light:Dark Conditions (A) Representative actograms for a *Bombus terrestris* forager on control food, or food containing 1 μg/L or 10 μg/L imidacloprid (IM). (B) Proportion of foragers that were arrhythmic (RS ≤1.5) in LD for each treatment. (C) Mean rhythmicity for either control foragers or those fed 1 or 10 μg/L imidacloprid, in LD conditions (*F*_2,58_ = 5.3, p = 0.008). (D) Mean locomotor activity for foragers in each treatment group in LD conditions, during the day (*F*_2,44_ = 6.7, p = 0.003) and the night (*F*_2,44_ = 5.4, p = 0.008). Each data point represents a single bee, n = 19–22 bees for each treatment group. Data are represented as mean ± SEM, ∗p ≤ 0.05, ∗∗p ≤ 0.01, tested via 1-way ANOVA with Tukey's multiple comparisons.

Exposure to field relevant concentrations of 1–10 μg/L imidacloprid disrupted the rhythmicity and quantity of locomotor activity in isolated foragers (Figure 1A). The rhythmicity statistic (RS) was calculated as a measure of rhythm strength (Levine et al., 2002), with RS > 1.5 by convention taken to indicate rhythmic behavior (Hodge and Stanewsky, 2008). In LD conditions, imidacloprid decreased mean rhythmicity (Figure 1C) and both 1 and 10 μg/L increased the proportion of foragers that were arrhythmic (Figure 1B), from 10% in control foragers to 36% and 67% respectively. Imidacloprid also reduced the total activity of foragers, with 1 μg/L reducing activity during both day and night and 10 μg/L reducing daytime activity (Figure 1D).

### Field-Relevant Concentrations of Imidacloprid Do Not Reduce Rhythmicity in Isolated Bumblebee Foragers in Constant Darkness

**Figure 2:**
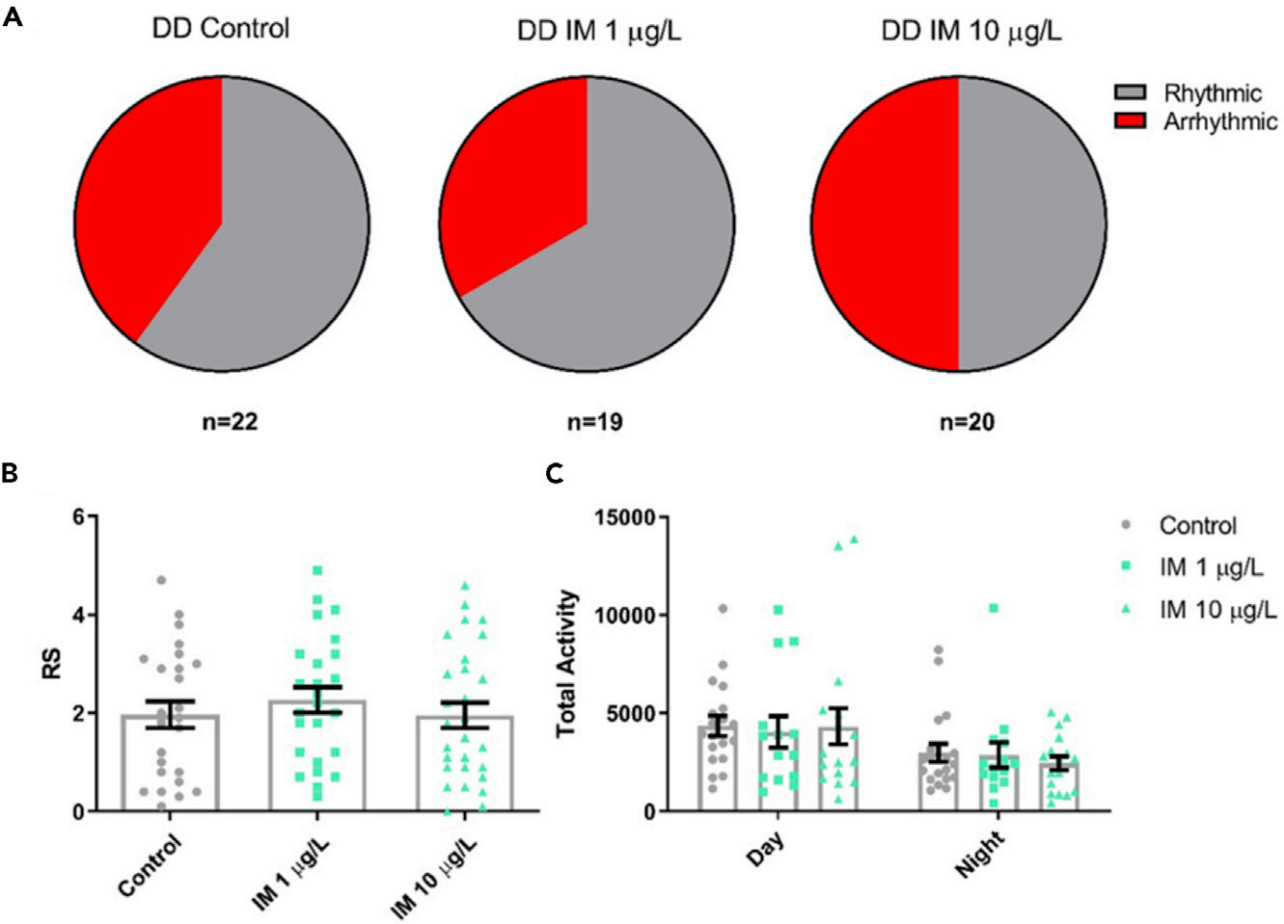
Field-Relevant Concentrations of Imidacloprid Do Not Reduce Rhythmicity in Isolated Bumblebee Foragers in Constant Darkness (A) Proportion of *Bombus terrestris* foragers that were arrhythmic (RS ≤ 1.5) in DD for foragers on control food or food containing 1 or 10 μg/L imidacloprid (IM). (B) Mean rhythmicity (RS) for foragers in each treatment group in DD conditions (*F*_2,47_ = 0.5, p = 0.637). (C) Mean locomotor activity for foragers in each treatment group in DD conditions, during the subjective day (*F*_2,47_ = 0.1, p = 0.947) and night (*F*_2,47_ = 0.6, p = 0.541). Each data point in the histograms represents a single bee, n = 14–22 bees for each treatment. Data are represented as mean ± SEM, tested via one-way ANOVA with Tukey's multiple comparisons.

Conversely, in DD, exposure to either concentration of imidacloprid had little effect on the forager's activity or rhythmicity (Figure 2). Foragers fed 1 or 10 μg/L imidacloprid had the same mean rhythmicity and levels of activity as control foragers (Figures 2B and 2C). The proportion of each population that was arrhythmic was also similar, with 40% of control foragers arrhythmic compared with 33% at 1 μg/L and 50% at 10 μg/L imidacloprid (Figure 2A).

### Field-Relevant Concentrations of Imidacloprid Increase Sleep in Isolated Bumblebee Foragers

**Figure 3:**
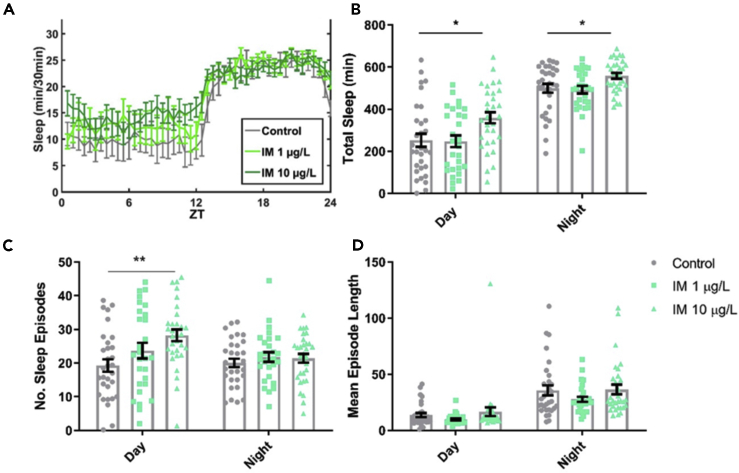
Field-Relevant Concentrations of Imidacloprid Increase Sleep in Isolated Bumblebee Foragers (A) Mean total sleep achieved for control *Bombus terrestris* foragers and those fed 1 μg/L (pale green line) or 10 μg/L (dark green line) imidacloprid (IM), per 30 min bin over the 24 h period. (B) Mean total sleep (min) for each treatment group in the day (*F*_2,87_ = 4.9, p = 0.010) and the night (*F*_2,87_ = 4.1, p = 0.019). (C) Mean number (No.) of sleep episodes initiated for each treatment group during the day (*F*_2,87_ = 5.4, p = 0.006) and the night (*F*_2,87_ = 0.490, p = 0.614). (D) Mean sleep episode length for each treatment group during the day (*F*_2,87_ = 1.7, p = 0.182) and the night (*F*_2,87_ = 1.5, p = 0.238). Each data point in the histograms represents a single bee, n = 28–31 bees for each treatment. Data are represented as mean ± SEM, ∗p ≤ 0.05, ∗∗p ≤ 0.01, tested via one-way ANOVA with Tukey's multiple comparisons.

Foragers who were fed 10 μg/L imidacloprid showed an increase in inactivity lasting longer than 5 min, usually taken as a proxy for sleep (Helfrich-Förster, 2018; Eban-Rothschild and Bloch, 2008), compared with controls. This was particularly notable during the day (Figures 3A and 3B) and is likely due to the increased number of daytime sleep episodes (Figure 3C) initiated by these foragers. The length of these sleep episodes was the same as in control foragers (Figure 3D).

### Field-Relevant Concentration of Imidacloprid Reduces Foraging Rhythmicity and Activity in Bumblebee Foragers within the Colony in 12 h:12 h Light:Dark Conditions

**Figure 4:**
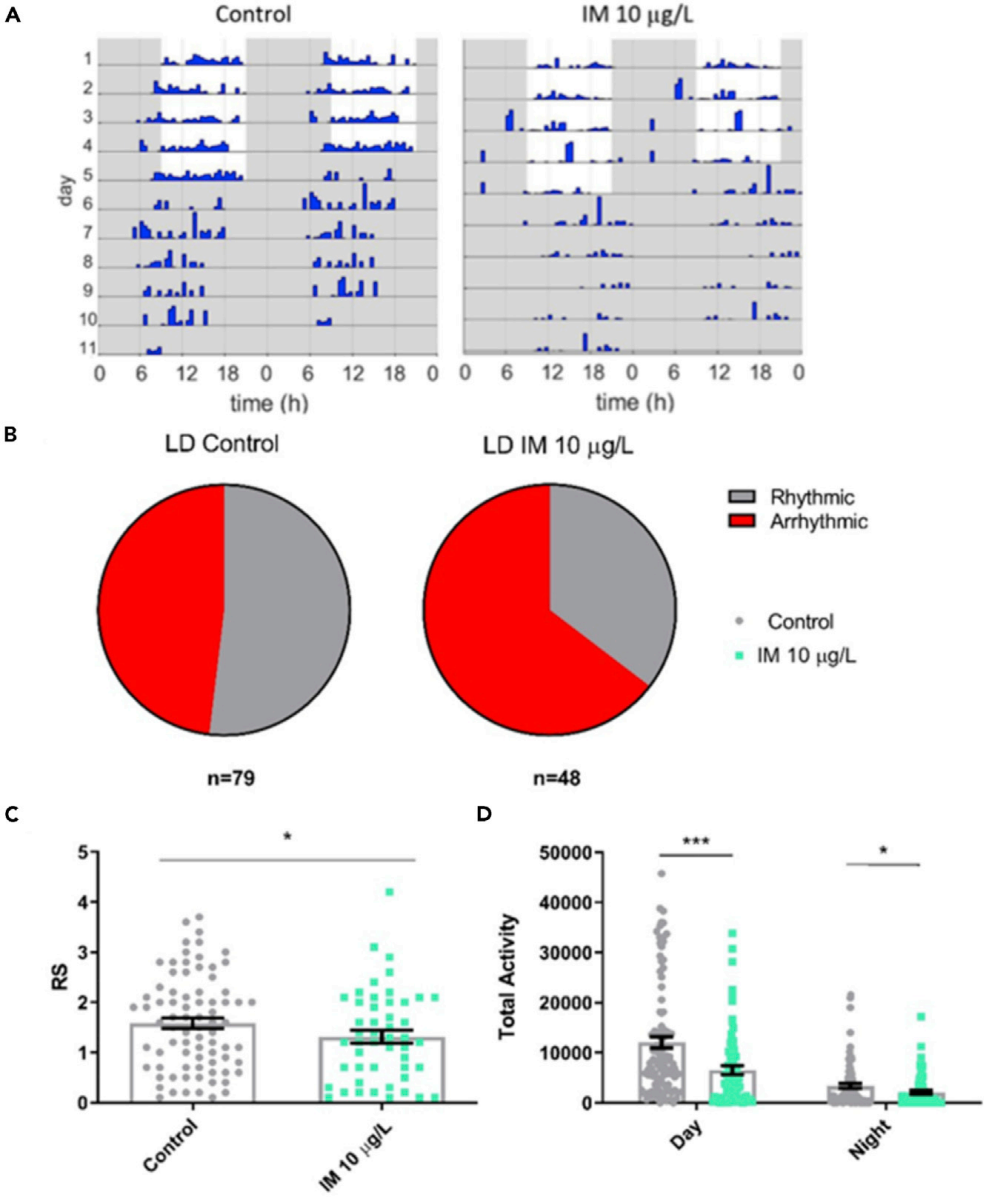
Field-Relevant Concentration of Imidacloprid Reduces Foraging Rhythmicity and Activity in Bumblebee Foragers within the Colony in 12 h:12 h Light:Dark Conditions (A) Representative actograms for a *Bombus terrestris* forager on control food and food containing 10 μg/L imidacloprid (IM). (B) Proportion of foragers that were arrhythmic in LD for each treatment group. (C) Mean rhythmicity for either control foragers or foragers fed 10 μg/L IM in LD (*t*_125_ = 2.0, p = 0.048). (D) Mean foraging activity for foragers in each treatment group in LD, during the day (*t*_170_ = 3.8, p < 0.001) and the night (*t*_170_ = 2.0, p = 0.042). Each data point in the histograms represents a single bee, n = 48–79 bees for each treatment for rhythmicity, n = 74–100 bees for each treatment for activity. Data are represented as mean ± SEM, ∗p ≤ 0.05, ∗∗∗p ≤ 0.001, tested via one-way ANOVA with Tukey's multiple comparisons.

**Figure 5:**
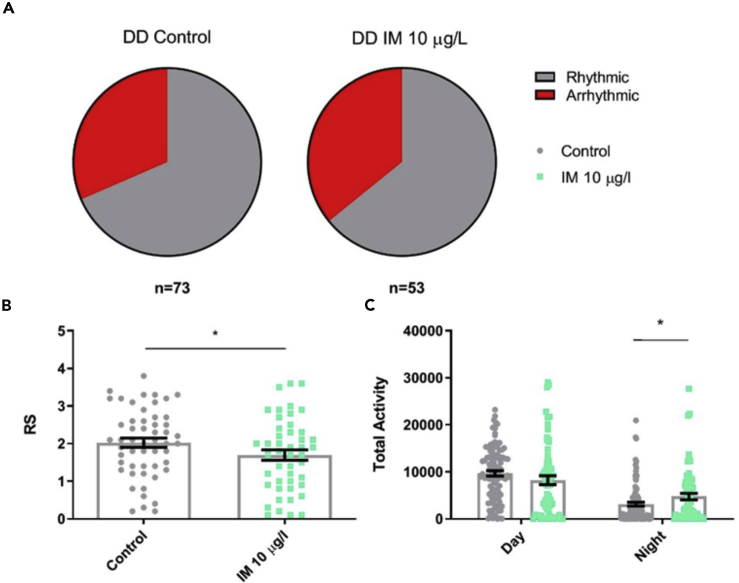
Imidacloprid Reduces Foraging Rhythmicity and Activity in Bumblebee Foragers within the Colony in Constant Darkness (A) Proportion of the *B. terrestris* foragers that were arrhythmic in DD on control food and food containing a field-relevant concentration of 10 μg/L imidacloprid (IM). (B) Mean rhythmicity for foragers in each treatment group in DD conditions (*t*_125_ = 2.2, p = 0.029). (C) Mean activity for foragers in each treatment group in DD conditions, during the subjective day (*t*_169_ = 1.6, p = 0.105) and night (*t*_114_ = −2.0, p = 0.043). Each data point in the histograms represents a single bee, n = 53–73 bees for each treatment for rhythmicity, n = 74–100 bees for each treatment for activity. Data are represented as mean ± SEM, ∗p ≤ 0.05, tested via one-way ANOVA with Tukey's multiple comparisons.

*B. terrestris* foragers in a full colony setting showed diurnal rhythms in foraging activity (Figures 4 and 5). This rhythmicity was decreased in foragers exposed to 10 μg/L imidacloprid in both LD (Figure 4) and constant darkness (Figure 5). In LD conditions, imidacloprid decreased mean rhythmicity of foragers (Figure 4C) and increased the proportion of foragers who were arrhythmic from 48% to 65% (Figure 4B). Imidacloprid decreased foraging activity for both daytime and night-time (Figure 4D).

### Imidacloprid Reduces Foraging Rhythmicity and Activity in Bumblebee Foragers within the Colony in Constant Darkness

In DD conditions, imidacloprid reduced mean rhythmicity for foragers (Figure 5B) and increased foraging activity during the subjective night (the 12 h that were dark during the entrainment period) (Figure 5C). The proportion of foragers that were arrhythmic for control and imidacloprid-exposed bees were similar in DD, 31% and 36% respectively (Figure 5A).

Therefore, field-relevant imidacloprid concentrations disrupted circadian rhythmicity and increased mistimed daytime inactivity or sleep in isolated *B. terrestris* foragers and reduced foraging rhythmicity for foragers in the colony. Foragers both in isolation and the colony showed that neonicotinoids cause a profound disruption of timing of activity with reduced daytime activity.

## Discussion

We report the effects of imidacloprid on pollinators, such as reduced locomotion and foraging activity, and go on to show that imidacloprid causes mistiming of these activities, increasing the proportion of foraging that occurs at night and increasing daytime inactivity. Imidacloprid has previously been shown to reduce locomotion in isolated *B. terrestris* (Cresswell et al., 2014), sweat bees like *Melipona quadrifasciata* (Tomé et al., 2012), and solitary bees like *Osmia bicornis* (Azpiazu et al., 2019), and to reduce in-nest activity in another bumblebee *B. impatiens* (Crall et al., 2018). We found that imidacloprid exposure also reduced the foraging activity within the colony, with foragers making fewer foraging trips in LD conditions. This is similar to previous laboratory studies, for example, imidacloprid exposure can cause reduced and less efficient foraging for sucrose solution in *B. impatiens* (Muth and Leonard, 2019) and mixed exposure to thiamethoxam and clothianidin has been shown to reduce foraging effort for both sucrose solution and pollen in *B. terrestris* (Fauser-Misslin et al., 2014). Other field-based studies have shown a reduction in pollen foraging efficiency in *B. terrestris* following exposure to imidacloprid (Gill and Raine, 2014; Gill et al., 2012), although with no reduction in the quantity of nectar collected per foraging bout. Neonicotinoid exposure appears to interfere with foraging efficiency, limiting the capacity of bees to handle flowers, carry out buzz pollination, and changing their flower preferences (Whitehorn et al., 2017; Stanley and Raine, 2016; Gill and Raine, 2014). Our study, and the other laboratory studies mentioned, focus on the foraging motivation within the colonies, which also appears to be reduced. This could in part be driven by the apparent appetite suppression that imidacloprid can cause, with 10 μg/L shown to decrease feeding by 30% in *B. terrestris* (Cresswell et al., 2012). Or it could be a result of reduced mobility, an effect that has been observed for imidacloprid exposure (Williamson et al., 2014).

We also show that field-relevant doses of imidacloprid impact locomotor and foraging rhythmicity. Imidacloprid reduced the rhythmicity of daily activity in foragers in LD conditions, suggesting that the neonicotinoid may be interfering with light input into the clock. Light signaling from the visual circuit and the Hofbauer-Buchner eyelet (another light sensing organ) to the clock neurons is dependent on nAChRs, so this is a potential route for disruption (Helfrich-Förster et al., 2002; Muraro and Ceriani, 2015). In *Drosophila*, imidacloprid appears to affect circadian rhythmicity and to prevent day/night differences in arborization and PDF accumulation in the small lateral-ventral neurons (sLNv) or pace making neurons of the clock, which receive light input from the Hofbauer-Buchner (HB) eyelet (Tasman et al., 2020). Foraging rhythmicity in DD was also reduced, possibly due to the reduction in entrainment during the LD stage of the experiments. Previous work showed that nAChR agonists can directly stimulate clock neurons in a cockroach *Rhyparobia maderae* causing increased calcium influx through voltage-gated calcium channels (Baz El et al., 2013). Likewise, nAChR agonists increase calcium influx of *Drosophila* PDF-releasing lateral ventral neurons (LNvs) (Wegener et al., 2004; Lelito and Shafer, 2012). Conversely nAChR antagonists block spike-dependent nAChR synaptic signaling required for rhythmic LNv activity (McCarthy et al., 2011). Work in another cockroach, *Periplaneta americana,* showed that neonicotinoids can also act on the thoracic ganglia, which control motor function in insects (Tan et al., 2007). Thus, neonicotinoids may be acting directly through multiple neurons regulating both locomotion and circadian regulation of locomotion, causing activation and/or depolarization block and hence compromising rhythmic foraging activity. In addition to direct classical pharmacological drug-receptor interactions, neonicotinoid exposure changes expression of hundreds of genes in worker bumblebees (*B. impatiens*), including genes that are involved in locomotion (Tan et al., 2007).

The reduction in rhythmicity observed for foragers in LD conditions and within the colony suggest that the effect of imidacloprid on rhythmicity cannot be mitigated by strong zeitgebers (i.e., time-givers or entrainment signals) such as light or social cues (Jurgen Stelzer et al., 2010; Bloch, 2010). This may imply that the reduction in foraging rhythmicity observed for bumblebee colonies exposed to imidacloprid in the laboratory are likely to reflect deleterious consequences in the field, as is the case for reductions in foraging activity (Gill and Raine, 2014; Muth and Leonard, 2019). A disruption of the clock in foragers may further reduce their foraging efficiency as they will not be able to form the time-memories required to accurately visit different flowers (van Nest et al., 2018; Bloch et al., 2017). The clock also feeds into the sun-compass navigation pathway that foragers use to navigate (Beer et al., 2018). Neonicotinoids have previously been shown to reduce homing ability in bees (Tosi et al., 2017; Fischer et al., 2014), although Stanley et al. found no effect (Stanley et al., 2016). If navigation is affected, then disruption to the clock could be a contributing factor to this. The reduced foraging motivation observed here is likely to compound the reduced pollen foraging efficiency observed in the field (Gill and Raine, 2014; Gill et al., 2012; Feltham et al., 2014) and is likely to reduce the capacity of the colony to grow and reproduce. Reduced feeding and foraging are associated with less brood production (Laycock et al., 2012) and smaller colonies, which are less resilient and less likely to produce queens (Whitehorn et al., 2012). The colony also responds to reduced foraging by producing and sending out more foragers, increasing forager mortality, and resulting in fewer workers to carry out in-nest tasks such as brood care (Gill et al., 2012).

Field-relevant concentrations of imidacloprid also increased sleep or total inactivity, with 10 μg/L increasing daytime sleep or total inactivity in foragers, hence the reduction in daytime locomotor and foraging activity observed. It is common practice to use 5 min of continuous inactivity as a proxy for sleep, and this practice has been validated (Helfrich-Förster, 2018; Eban-Rothschild and Bloch, 2008; Hendricks et al., 2020). However, as imidacloprid can cause immobilization, in this case it is not possible to differentiate between sleep and immobilization (Williamson et al., 2014). There are possible routes for either effect. The mushroom bodies, which are known to regulate the sleep/wake cycle in insects, contain groups of both wake- and sleep-promoting Kenyon neurons and signal via nAChRs to the mushroom body output neurons, which are also important for sleep regulation (Pitman et al., 2006; Helfrich-Förster, 2018; Barnstedt et al., 2016). Sleep-promoting neurons have been shown to be specifically activated by nAChRs (Aso et al., 2014), providing a possible route for imidacloprid to induce sleep. Furthermore, whole-cell patch-clamp recordings from honeybee Kenyon neurons from the mushroom body showed that they were directly stimulated by imidacloprid (Moffat et al., 2016). However, as mentioned above, the thoracic ganglia and motor neurons are also nicotinic, allowing possible stimulation or depolarizing block of these. Imidacloprid has been shown to cause immobilization, increased time spent upside down, and difficulty in motor tasks in bees (Williamson et al., 2014). Either way, increased immobility or sleep in the colony reduces opportunities for foraging and potentially for in-nest tasks such as brood care.

Neonicotinoids reduce activity and foraging motivation in pollinators (Muth and Leonard, 2019; Fauser-Misslin et al., 2014; Gill and Raine, 2014; Gill et al., 2012). Here we demonstrate that very low field-relevant concentrations of neonicotinoids disrupt rhythmicity of foraging activity as well as increasing daytime immobility, further reducing the opportunities for bees to forage and pollinate and having knock-on effects on circadian and sleep-regulated physiological and behavioral processes in the bee. This is likely to have a detrimental effect on colony fitness in the field as well as reducing the yield of crops and wild plants reliant on bee pollination. Furthermore, we establish a number of highly sensitive (down to 1 ppb neonicotinoids) high-throughput behavioral assays for measuring the detrimental sublethal effects of insecticides on pollinators.

### Limitations of the Study

A key limitation of the study is the inability to differentiate between sleep and inactivity. Another is that the dose consumed by each bee was not quantified exactly, although bees were allowed to feed freely from sugar syrup with a field-realistic concentration of imidacloprid and the dose estimated from the average quantity consumed.

### Resource Availability

#### Lead Contact

Further information and requests for resources and reagents should be directed to and will be fulfilled by the lead contact, Dr. James Hodge (james.hodge@bristol.ac.uk).

#### Materials Availability

No novel materials or reagents were used for these experiments.

#### Data and Code Availability

Original data have been deposited to Mendeley Data: https://doi.org/10.17632/m8pykxzkyb.1.

## Methods

All methods can be found in the accompanying Transparent Methods supplemental file.

## Supplemental Information

Document S1. Transparent Methods

## Footnotes

Supplemental Information can be found online at https://doi.org/10.1016/j.isci.2020.101827.
