# Supplementary material for: The Neonicotinoid Insecticide Imidacloprid Disrupts Bumblebee Foraging Rhythms and Sleep
Source: iScience. 2020 Nov 20;23(12):101827. doi: 10.1016/j.isci.2020.101827 (PMC7710657; doi:10.1016/j.isci.2020.101827)
Supplement: Document S1. Transparent Methods [file mmc1.pdf]

iScience, Volume *23*

Supplemental Information

The Neonicotinoid Insecticide Imidacloprid Disrupts Bumblebee Foraging Rhythms and Sleep

Kiah Tasman, Sean A. Rands, and James J.L. Hodge

TRANSPARENT METHODS

Further information and requests for resources should be directed to Dr James JL Hodge (james.hodge@bristol.ac.uk).

Bombus terrestris colonies

Bombus terrestris audax colonies (Biobest), containing cotton wool and between 80-100 workers, were ordered through Agralan (#BB121040-CF1). A total of 10 colonies were used, four for the isolated forager experiments and six for the colony wide experiments. They were maintained at 21°C, 12h:12h light: dark (LD) with lights on at 9am and off at 9pm, with a 30 mins dawn/dusk period where lights were at 50%. Colonies were provided with Biogluc® (Biobest) *ad lib* in the foraging arena and 1 tablespoon (15 ml) pollen (Agralan #BB008513) every 5 days which was introduced into the nest box. Pollen was not provided more regularly during the experiment in order to prevent any food-based entrainment and to keep conditions the same during the LD section as in the DD section. During constant darkness (DD) the set up could not be accessed and pollen could not be provided in order to prevent light contamination. Imidacloprid (PESTANAL Sigma-Aldrich #37894), was administered *via* the Biogluc®, at field relevant concentrations of either 1 or 10 µg/L[23]. Imidacloprid was taken from a 100,000 µg/L ddH_2O frozen stock solution, mixed into 10ml ddH_2O and then into 990 ml Biogluc using a magnetic stirrer and flea. As the actual concentration was not tested, the stated concentrations of 1 and 10 µg/L are nominal concentrations. For both concentrations, bees consumed approximately 0.2 ml of sucrose solution per day, resulting in approximate doses of 0.16-0.2 and 1.6-2 ng per bee (calculated by dividing the quantity of solution consumed over the ten days period by ten and by the number of bees). Colonies were attached via a clear plastic tube (15 mm diameter and 200 mm length), to a foraging arena (1000 × 500 × 500 mm) purpose built by the University of Bristol Mechanical Workshop out of UV-transmitting acrylic. A wide ramp from the entrance to the foraging arena to the floor of the arena was built from card and duct tape to ensure that foragers could return to the nest box in darkness, when they cannot fly (Reber et al., 2015).

Circadian rhythm analysis for isolated foragers

For the collection of circadian and sleep data for bumblebees, a system was set up using the Locomotor Activity Monitor (LAM16, TriKinetics Inc, USA) (Beer et al., 2016). Large foragers, of around 15-16 mm in length were collected from the foraging arena and loaded into tubes (PGT16x100). Only foragers who were seen coming out of the colony to feed on the feeder were selected. A total of 30 foragers were taken from each colon, ten per treatment group and put into the LAM set up together. This was repeated for between three and four colonies (three colonies for LD data, four for sleep and DD). Only those foragers who were still active at the end of the study period were used for analysis. At one end of the tube was a rubber cap (CAP16-Black) with a 1 g silica packet glued inside to control the humidity. At the other end, a 15 mL Falcon tube with a small whole drilled near the base was attached. This contained Biogluc® with or without neonicotinoids, allowing bees to feed *ad libitum* throughout the experiment. The tubes were loaded into the LAM and monitored for five days in LD and five days in DD conditions. In the monitor an infrared beam crossed the diameter of the centre of the tube and every beam break was counted in real time by a host computer. Each beam break was counted as a single activity bout, allowing the total activity for each bee to be summed per day and per 30 mins bin for circadian analysis. For each bee, the rhythmicity statistic (RS), period length and total daily activity levels were then calculated. The rhythmicity statistic was calculated using conventional autocorrelation analysis (Levine et al., 2002). First, the data were filtered, using a low-pass Butterworth filter to remove any periodicities of less than four hours. The dataset was then paired with itself, gradually moved out of register with itself and the correlation coefficient plotted. At 0 hour, the two data sets are identical, and then, for rhythmic data, they return to high correlation approximately every 24 hours. The value of the third peak in the auto-correlogram is reported as the rhythmicity index (RI), a statistical representation of the rhythmicity of the bee's activity. The RS is calculated as the ratio of this RI to the value of the 95% confidence line. This analysis was carried out using *Flytoolbox* (Levine et al., 2002) in MATLAB (MATLAB and Statistics Toolbox Release 2015b). Conventionally, an RS >2 is rhythmic, an RS of 1.5-2 is weakly rhythmic and an RS <1.5 is arrhythmic (Hodge and Stanewsky, 2008). The day and night activity levels for each bee were calculated using the *daynight* program (Julienne et al., 2017) in MATLAB. The individual bee's period length was used to split the activity data into subjective days and nights and then the activity counts for each were summed. The mean daily activity in daytime and night-time for the five days was then calculated.

Sleep analysis for isolated foragers

Sleep is defined as any inactivity lasting more than five minutes, as has been done in previous studies (Beyaert et al., 2012, Nagari et al., 2019, Eban-Rothschild and Bloch, 2015). Sleep analysis was carried out on the five days of LD (Buhl et al., 2019), using activity data that had been summed into both 1 min and 30 mins bins. Analysis was performed using the Sleep and Circadian Analysis MATLAB Program (SCAMP (Donelson et al., 2012)). The mean total quantity of sleep per 30 mins bin was calculated and displayed. Total quantity of sleep for the day and night for each bee was quantified and the mean taken for the five days. Other sleep measures reported were the number of sleep episodes initiated during the day and night and the mean length of these episodes.

Foraging rhythmicity assay for bumblebees in the colony

Circadian rhythmicity within the colony was assayed using a micro radio frequency identification (RFID) setup (Molet et al., 2008, Stelzer and Chittka, 2010). Approximately 40 foragers were collected from the foraging arena of the colony, three colonies per treatment group. Only foragers who were seen coming out of the colony to feed on the feeder were selected. These were anaesthetised using CO_2 and an RFID tag (Microsensys GmbH mic3-TAG) was stuck to the centre of their thorax with superglue (Loctite). Foragers were then returned to the colony nest box. After a day for acclimatisation and recovery from the CO_2 exposure, recording began. An RFID reader (Microsensys GmbH iID®MAJAreadermodule4.1) was placed at the entrance to the foraging arena so that foragers had to pass through the reader to enter the arena. The data from the readers were collected on a host (Microsensys GmbH iID®HOSTtypeMAJA4.1), for a 10 days period; 5 days LD and 5 days DD, as in the LAM experiments above. The data were then summed into 30 mins bins, with each pass through the reader being counted as a single activity bout. These data were then analysed. Only those that showed foraging activity every day of the study period were used for analysis, ensuring that only foragers were assessed. For control groups this was 24, 27 and 29 bees per colony. For the treatment group this was 10, 17 and 21 bees per colony. Pollen was provided the day before recording began and on day 5. Biogluc was available *ad libitum* in the foraging arena and was either untreated for control groups or contained 10 µg/L imidacloprid for treated groups. Three colonies were tested for each treatment group.

Statistical Analysis

Analysis was carried out using a one-way ANOVA and the results displayed in figure legends. First the data was checked for normality using a Shapiro-Wilk test. The homogeneity of variance was also tested using Levene's test for equality of variances. Means were then compared using a two-way ANOVA to quantify the effect of both treatment and colony. Once colony effects were shown to be nonsignificant, colony was removed as a factor and a one-way ANOVA was carried out with *post hoc* pairwise comparisons using Tukey's multiple comparisons test. Statistical analysis was done in IBM SPSS Statistics 24. Graphs were created in GraphPad (Prism version 8.0.0). For all histograms, every data point was plotted with lines showing the mean ± standard error of the mean (SEM). Where *post hoc* tests were done, these were displayed as $p \leq 0.05$*, $p \leq 0.01$**, $p \leq 0.001$***, $p \leq 0.0001$****).

SUPPLEMENTAL REFERENCES

BEER, K., STEFFAN-DEWENTER, I., HÄRTEL, S. & HELFRICH-FÖRSTER, C. (2016). A new device for monitoring individual activity rhythms of honey bees reveals critical effects of the social environment on behavior. *Journal of Comparative Physiology A,* 202**,** 555-565.

BEYAERT, L., GREGGERS, U. & MENZEL, R. (2012). Honeybees consolidate navigation memory during sleep. *Journal of Experimental Biology,* 215**,** 3981-8.

BUHL, E., HIGHAM, J. P. & HODGE, J. J. L. (2019). Alzheimer's disease-associated *tau* alters *Drosophila* circadian activity, sleep and clock neuron electrophysiology. *Neurobiology of Disease,* 130**,** 104507.

DONELSON, N., KIM, E., SLAWSON, J., VECSEY, C., HUBER, R. & GRIFFITH, L. (2012). High-resolution positional tracking for long-term analysis of *Drosophila* sleep and locomotion using the "Tracker" program. *PloS One,* 7**,** e37250.

EBAN-ROTHSCHILD, A. & BLOCH, G. (2015). The colony environment modulates sleep in honey bee workers. *Journal of Experimental Biology,* 218**,** 404.

HODGE, J. J. & STANEWSKY, R. (2008). Function of the Shaw potassium channel within the *Drosophila* circadian clock. *PLoS One,* 3**,** e2274.

JULIENNE, H., BUHL, E., LESLIE, D. S. & HODGE, J. J. L. (2017). *Drosophila* PINK1 and parkin loss-of-function mutants display a range of non-motor Parkinson's disease phenotypes. *Neurobiology of Disease,* 104**,** 15-23.

LEVINE, J. D., FUNES, P., DOWES, H. B. & HALL, J. C. (2002) Signal analysis of behavioural and molecular cycles. BMC Neuroscience, 3, 1.

MOLET, M., CHITTKA, L., STELZER, R., STREIT, S., and RAINE, N. E. (2008) Colony nutritional status modulates worker responses to foraging recruitment pheromone in the bumblebee *Bombus terrestris*. *Behavioural Ecology and Sociobiology*, 62, 1919-1926.

NAGARI, M., GERA, A., JONSSON, S. & BLOCH, G. (2019). Bumble bee workers give up sleep to care for offspring that are not their own. *Current Biology,* 29**,** 3488-3493.e4.

REBER, T., VÄHÄKAINU, A., BAIRD, E., WECKSTRÖM, M., WARRANT, E. & DACKE, M. (2015). Effect of light intensity on flight control and temporal properties of photoreceptors in bumblebees. *Journal of Experimental Biology,* 218**,** 1339.

STELZER, R. J. & CHITTKA, L. (2010). Bumblebee foraging rhythms under the midnight sun measured with radiofrequency identification. *BMC Biology,* 8**,** 93.
